# Pulmonary toxicity of tungsten trioxide nanoparticles in an inhalation study and an intratracheal instillation study

**DOI:** 10.1002/1348-9585.12367

**Published:** 2022-11-10

**Authors:** Takashi Marui, Taisuke Tomonaga, Hiroto Izumi, Yukiko Yoshiura, Chinatsu Nishida, Hidenori Higashi, Ke‐Yong Wang, Miyako Shijo, Masaru Kubo, Manabu Shimada, Yasuo Morimoto

**Affiliations:** ^1^ Department of Occupational Pneumology, Institute of Industrial Ecological Sciences University of Occupational and Environmental Health Fukuoka Japan; ^2^ Center for Stress‐related Disease control and Prevention University of Occupational and Environmental Health Fukuoka Japan; ^3^ Department of Respiratory Medicine University of Occupational and Environmental Health Fukuoka Japan; ^4^ Department of Environmental Health Engineering, Institute of Industrial Ecological Sciences University of Occupational and Environmental Health Japan Fukuoka Japan; ^5^ Shared‐Use Research Center, School of Medicine University of Occupational and Environmental Health Fukuoka Japan; ^6^ Department of Advanced Science and Engineering Graduate School of Advanced Science and Engineering, Hiroshima University Hiroshima Japan

**Keywords:** chronic effect, inhalation, intratracheal instillation, nanoparticle, persistent inflammation, tungsten trioxide

## Abstract

**Objectives:**

We conducted inhalation and intratracheal instillation studies in order to examine the effects of tungsten trioxide (WO_3_) nanoparticles on the lung, and evaluated whether or not the nanoparticles would cause persistent lung inflammation.

**Methods:**

In the inhalation study, male 10‐week‐old Fischer 334 rats were classified into 3 groups. The control, low‐dose, and high‐dose groups inhaled clean air, 2, and 10 mg/m^3^ WO_3_ nanoparticles, respectively, for 6 h each day for 4 weeks. The rats were dissected at 3 days, 1 month, and 3 months after the inhalation, and the bronchoalveolar lavage fluid (BALF) and lung tissue were examined. In the intratracheal instillation study, male 12‐week‐old Fischer 334 rats were divided into 3 subgroups. The control, low‐dose, and high‐dose groups were intratracheally instilled 0.4 ml distilled water, 0.2, and 1.0 mg WO_3_ nanoparticles, respectively, dissolved in 0.4 ml distilled water. The rats were sacrificed at 3 days, 1 week, and 1 month after the intratracheal instillation, and the BALF and lung tissue were analyzed as in the inhalation study.

**Results:**

The inhalation and instillation of WO_3_ nanoparticles caused transient increases in the number and rate of neutrophils, cytokine‐induced neutrophil chemoattractant (CINC)‐1, and CINC‐2 in BALF, but no histopathological changes or upregulation of heme oxygenase (HO)‐1 in the lung tissue.

**Conclusion:**

Our results suggest that WO_3_ nanoparticles have low toxicity to the lung. According to the results of the inhalation study, we also propose that the no observed adverse effect level (NOAEL) of WO_3_ nanoparticles is 2 mg/m^3^.

## 1 INTRODUCTION

A nanoparticle is defined as a particle with the length of all external dimensions in the range of approximately 1 to 100 nm, where the lengths of the longest and the shortest axes of the particle do not differ significantly.^1^ Most nanomaterials, including nanoparticles, have newly added functions. It is expected that the application of nanomaterials in various areas, including agriculture and food industry, drinking water systems, renewable energy systems, high speed optical computers, and health and medical solutions, will be expanded in the future because they could provide ways to solve problems and challenges in different branches of society, and could help us achieve the Sustainable Development Goals (SDGs).^2^

Tungsten trioxide (WO_3_) nanoparticles are a yellow powder. The technical application of WO_3_ nanoparticles is advanced in the area of visible‐light photocatalysts, and it is thought that they could contribute to removing house dust or to forming a sterile environment. In spite of its variety of uses, the toxicity of WO_3_ nanoparticles, especially to humans, has not been studied fully. In the process of the hazards caused by chemical substances, including nanomaterials, being inhaled into the lung, it is suspected that persistent inflammation in the alveolus can lead to pulmonary fibrosis and lung cancers. Persistent inflammation in the alveolus is involved in free radical damage to the lung, which can provoke abnormal tissue repair, repeated genetic and epigenetic aberrations of alveolar epithelial cells, and finally the occurrence of fibrosis and tumors.^3^ Persistent inflammation caused by silica and asbestos, for example, are related to the occurrence of tumors.^4^, ^5^ In order to investigate the lung toxicity of nanoparticles, it is important to find out whether they cause persistent inflammation.

We examined pulmonary inflammation in rat induced by WO_3_ nanoparticles by an inhalation study, which has similarities to human exposure, and by an intratracheal instillation study, which allows relatively large doses if the size of the agglomeration of nanomaterials is maintained at a respirable level.

## 2 METHODS

### 2.1 WO_3_ nanoparticles

The WO_3_ nanoparticles used in the inhalation study and intratracheal instillation study were provided by Toshiba Material Co., Ltd. The geometric primary particle size, analyzed by a transmission electron microscope (TEM), was 19 ± 1 nm (“±” means geometric standard deviation; the same applies hereinafter). The surface area was 24.0 m^2^/g, as calculated by the Brunauer–Emmett–Teller method using nitrogen adsorption measurement.^6^ The average of the DLS of the agglomeration in 2.5 mg of WO_3_ nanoparticles in 1 mL water was 45.56 nm.

### 2.2 Animals

Male 9‐week‐old Fischer 334 rats were purchased from the Charles River Laboratories Japan, Inc., and reared in the Laboratory Animal Research Center of the University of Occupational and Environmental Health with free access to food and water. The animals were acclimated to the environment for 1 week after being introduced to the facility so that the intermediate time of the inhalation exposure period (the twelfth week) equaled that of the intratracheal instillation. All procedures, including the handling of rats, were done in accordance with the guidelines described in the Japanese Guide for the Care and Use of Laboratory Animals as passed by the Animal Care and Use Committee, University of Occupational and Environmental Health, Japan (Permit No. AE17‐010 (inhalation study); AE11‐012 (intratracheal instillation study)).

### 2.3 Inhalation study

**FIGURE 1:**
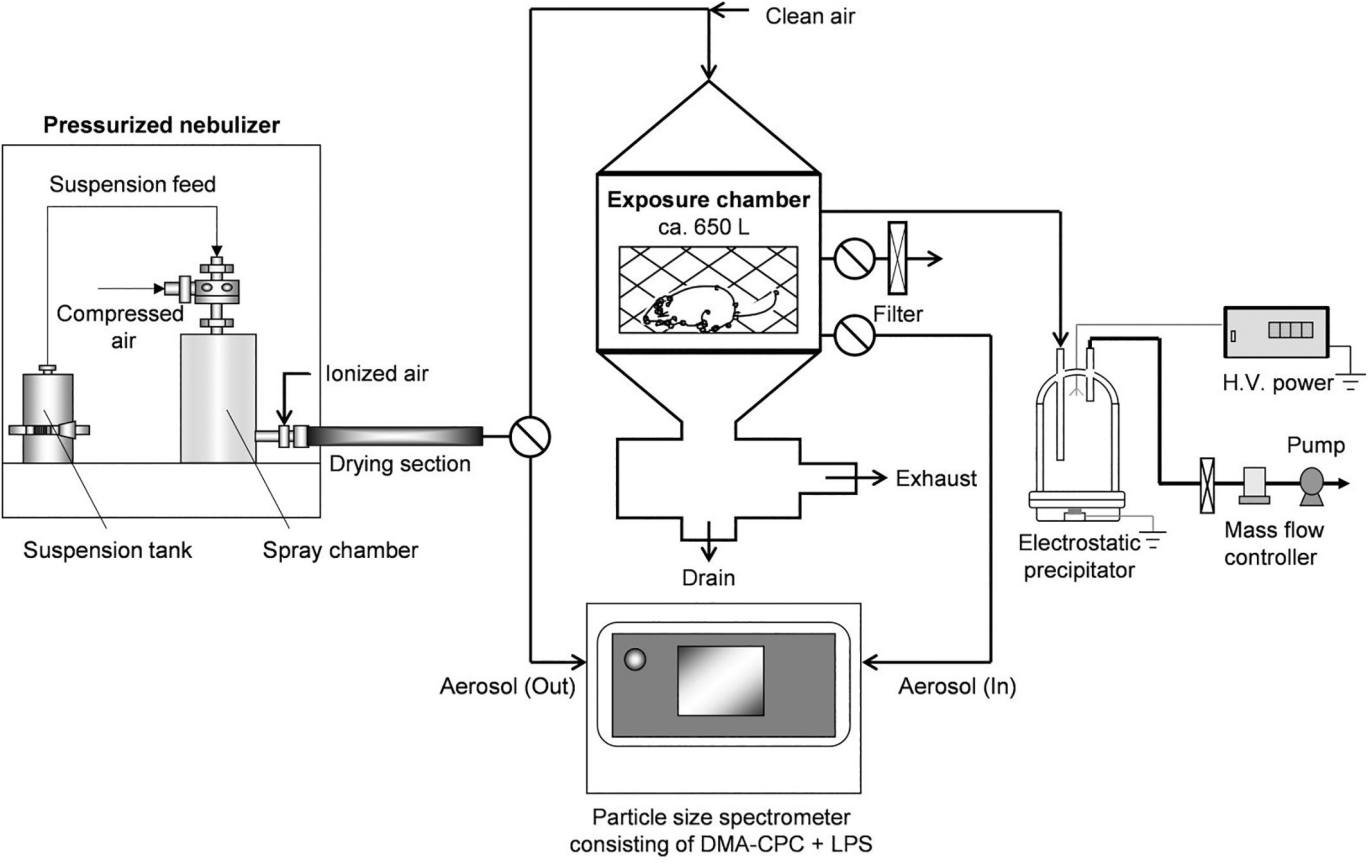
Schematic diagram of the inhalation test system

The inhalation study was conducted by an inhalation test system which was similar to that used in our previous studies.^7^, ^8^ A schematic diagram of the system is shown in Figure 1. The system was primarily comprised of a pressurized nebulizer (Nanomaster, JSR Corp.), an ionizer (SJ‐M, Keyence Corp.), a drying section, a stainless‐steel whole‐body exposure chamber with rat cage, an electrostatic precipitator, and a wide range particle size spectrometer (Model 1000XP WPS, MSP Corp.) that consisted of a differential mobility analyzer and a condensation particle count (DMA‐CPC) system and laser particle spectrometer (LPS) for in‐line monitoring.

The WO_3_ nanoparticle suspension was sprayed by the nebulizer with pressurized clean air at a flow rate of 40 L/min and with a suspension at a flow rate of 0.8 ml/min. The sprayed droplets were heated to 100°C in a spray chamber to induce rapid evaporation.^8^ The droplets were electrically neutralized by mixing 15 L/min air containing bipolar ions supplied by the ionizer to reduce aerosol particle wall loss in the following tube caused by electrostatic forces. The aerosol was diluted with clean air at a flow rate of 45 L/min prior to entering the exposure chamber to achieve a required total airflow rate of 100 L/min.

The size and number concentration of the aerosol particles inside and outside the exposure chamber were analyzed using the particle size spectrometer. The size distribution was measured every 30 min per day. The average and standard deviations of the aerosol particle size during the entire test period were calculated from the 260 geometric mean diameter of each size distribution. The aerosol particles were collected on a TEM grid by the electrostatic precipitator for a TEM measurement (TEM, JEM‐2010, JEOL). The mass concentration of the aerosol in the chamber was determined using a gravimetric method. The aerosol was admitted through fibrous filters for 30 min, and the collected particles were weighed. The mass concentration was calculated by dividing the amount of collected aerosol particles by the total volume of sampling air. The measurement of mass concentration was conducted three times per day. The average and standard deviation of the mass concentration was calculated from all the mass concentrations during the entire test period.

### 2.4 Inhalation exposure protocol

The group of 10‐week‐old rats was divided into 3 subgroups, named the control group, the low‐dose group, and the high‐dose group, respectively. The subgroups, consisting of 15 rats each, inhaled the following air or aerosol in the exposure chambers for 6 h on each day, for 5 days in each week of the inhalation test (4 weeks): the control group inhaled clean air; the low‐dose group inhaled the aerosol of WO_3_ nanoparticles at a concentration of 2 mg/m^3^; and the high‐dose group inhaled the aerosol of WO_3_ nanoparticles at a concentration of 10 mg/m^3^. The amounts of nanoparticles that were dispersed in distilled water were adjusted by the day. Rats were dissected at 3 days, 1 and 3 months after the 4 weeks of inhalation.

### 2.5 Intratracheal instillation study

As in the inhalation study, the group of 12‐week‐old rats was divided into 3 subgroups: the control group, the low‐dose group, and the high‐dose group, respectively. The WO_3_ nanoparticles were suspended in 0.4 ml distilled water and administered into the tracheas of the low‐dose group and high‐dose group animals in a single dose. The amounts of instilled particles were 0.2 mg in the low‐dose group and 1.0 mg in the high‐dose group. Similarly, 0.4 ml distilled water was intratracheally instilled to the rats in the control group. The rats were sacrificed at 3 days, 1 week, and 1 month after the intratracheal instillation.

### 2.6 Process of dissection, lung tissue, and bronchoalveolar lavage fluid (BALF)

In the dissections, the left lungs were inflated with saline under a pressure of 25 cm water, and the BALF was collected. After the collections of BALF ranging from 10 to 14 ml, the left lungs were fixed by 10% of formaldehyde to evaluate the inflammation histopathologically. The right lungs were divided into crumbs ranging from 40 to 60 mg and homogenized to perform analyses of heme oxygenase (HO)‐1 in the lung tissue.

The BALF was centrifuged at 400 *g* at 4°C for 15 min and divided into the supernatant fluid and the pellet. The fluid was used to perform manual analyses of the concentration of cytokine‐induced neutrophil chemoattractant (CINC)‐1, CINC‐2, and the activity of lactate dehydrogenase (LDH).

### 2.7 Cell analysis in BALF with cytospin

The pellet was washed with a suspension of PMN Buffer (137.9 mM NaCl, 2.7 mM KCl, 8.2 mM Na_2_HPO_4_, 1.5 mM KH_2_PO_4_, and 5.6 mM C_6_H_12_O_6_) and centrifuged at 1500 rpm at 4°C for 15 min. After the removal of the supernatant fluid, the pellet was resuspended with 1 ml of PMN Buffer. The number of cells in the suspension was calculated by blood cell counters, after which the suspension was smeared on slide glasses, using cytospin, fixed, and dyed with Diff‐Quik (Sysmex Corporation). The number of neutrophils was counted by microscopic observation after the slide glasses dried.

### 2.8 Measurement of LDH activity and chemokines in BALF, and HO‐1 in lung tissue

The LDH activity in the BALF was measured by a Cytotoxicity Detection Kit^PLUS^ (LDH) (F. Hoffmann‐La Roche, Ltd.). The enzyme‐linked immunosorbent assays (ELISAs) for CINC‐1 and CINC‐2 in the BALF, and HO‐1 in the lung tissue were performed using a Rat CXCL1/CINC‐1 Quantikine ELISA Kit (#RCN100), a Rat CXCL3/CINC‐2 alpha/beta Quantikine ELISA Kit (#RCN200) (both: R&D Systems), and a HO‐1 (rat), ELISA kit (#ADI‐EKS‐810A) (Enzo Life Sciences), respectively.

### 2.9 Histopathology

The left lobes were embedded in paraffin and cut into small sections. They were stained with hematoxylin and eosin and examined histopathologically.

### 2.10 Statistical analysis

Dunnett's test was applied where appropriate to determine individual differences using a computer statistical package, SPSS (IBM). A *P*‐value of .05 was chosen as the significance level when there was a significant difference. In addition to the Dunnett's test, Spearman's rank correlation coefficient was used when the correlation between the chemokines and the number of neutrophils in the BALF was analyzed.

## 3 RESULTS

### 3.1 Characterization of WO_3_ nanoparticles in the inhalation study

**FIGURE 2:**
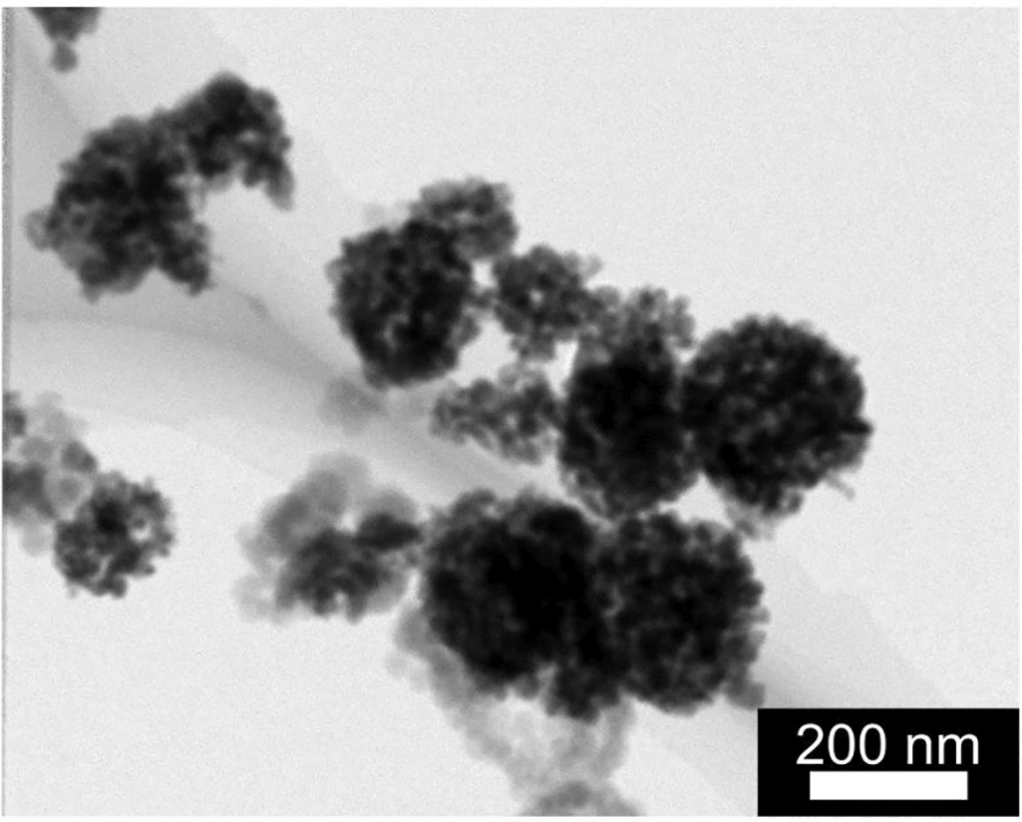
TEM image of WO_3_ nanoparticles in the chamber of the high‐dose group inhalation test.

Figure 2 shows the TEM image of the WO_3_ nanoparticles in the chamber of the high‐dose group inhalation test. The averages of agglomeration size in the chamber in the inhalation study were 125 ± 7 nm in the low‐dose group and 152 ± 6 nm in the high‐dose group, respectively. The averages of mass concentration were 2.1 ± 0.2 mg/m^3^ in the low‐dose group and 10 ± 1 mg/m^3^ in the high‐dose group, respectively.

### 3.2 Cell analysis in BALF

**FIGURE 3:**
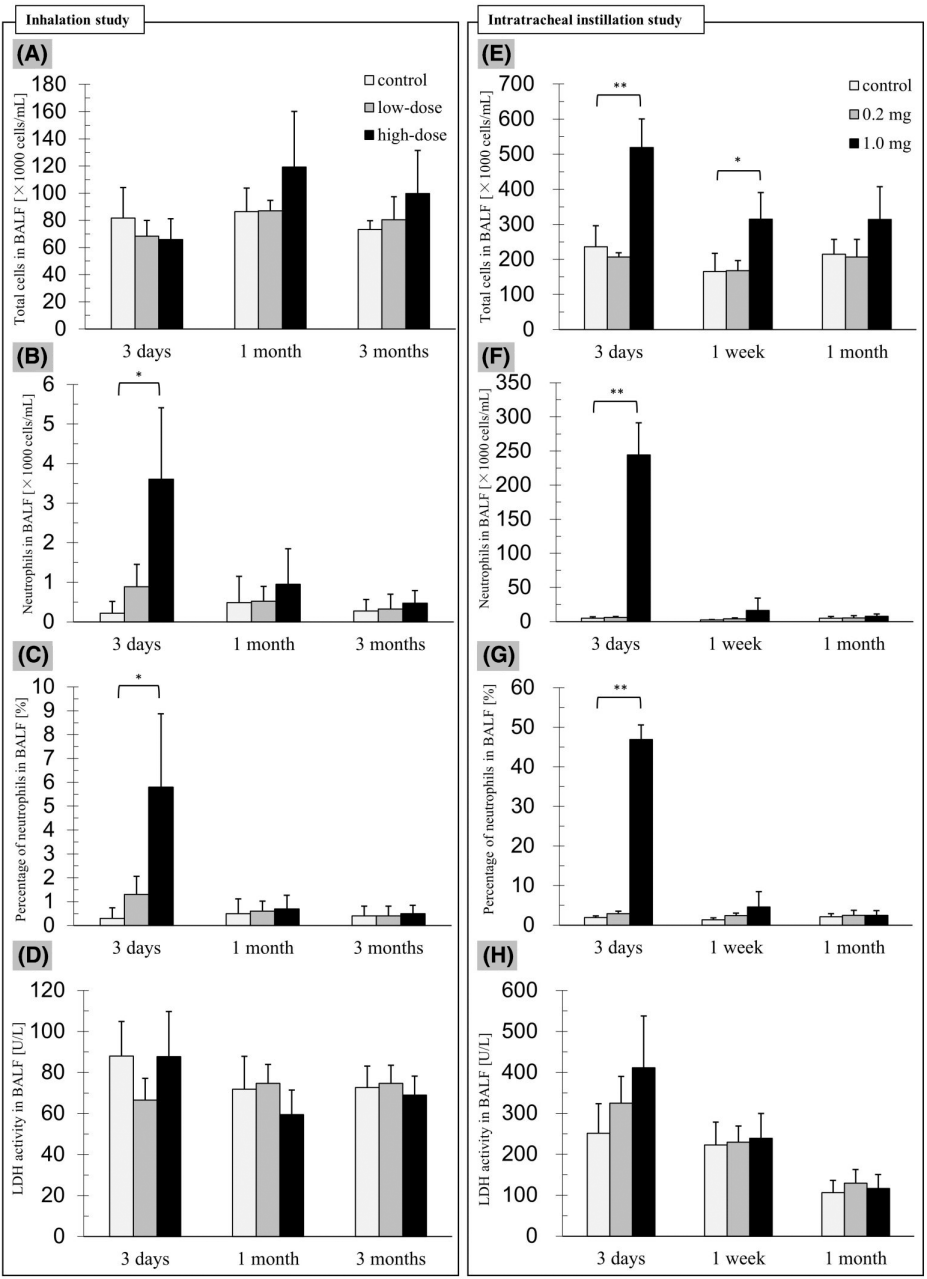
The cell analyses in the BALF. Asterisks indicate significant differences compared with the control groups (*P < .05, **P < .01). In both the inhalation and intratracheal instillation studies, the numbers (B, F) and rates of neutrophils (C, G) in the high‐dose groups were significantly higher at 3 days after exposures, but not at the other time points. In the intratracheal instillation study, the statistical analysis showed a significant increase in the total cell count (E) in the BALF in the high‐dose group compared with the control‐group at 3 days after the intratracheal instillation. On the other hand, in the inhalation study, the total cell count (A) did not increase significantly in any of the groups in every phase. In the LDH release in the BALF (D, H), there were no significant differences between any of the groups at each time point in either study.

Inhalation study: In comparison with the control group, the number (Figure 3B) and rate (Figure 3C) of neutrophils in the BALF were significantly higher in the high‐dose group dissected at 3 days after the exposure (the number: *P* = .018; the rate: *P* = .039). However, the statistical analysis showed no significant difference between the control group and the low‐dose group at 3 days or between all the groups at 1 and 3 months. The total cell count (Figure 3A) and LDH release (Figure 3D) did not increase significantly in any of the groups at any of the time points.

Intratracheal instillation study: Dunnett's test showed a statistical increase in total cell count (Figure 3E), neutrophil count (Figure 3F), and percentage of neutrophils (Figure 3G) in the BALF in the high‐dose group compared with the control‐group at 3 days after the intratracheal instillation (both: *P* < .001). In contrast, there were no significant differences between all the groups at 1 month. No significant increases in LDH release (Figure 3H) were observed in any of the groups at any of the time points.

### 3.3 CINC concentration in BALF

**FIGURE 4:**
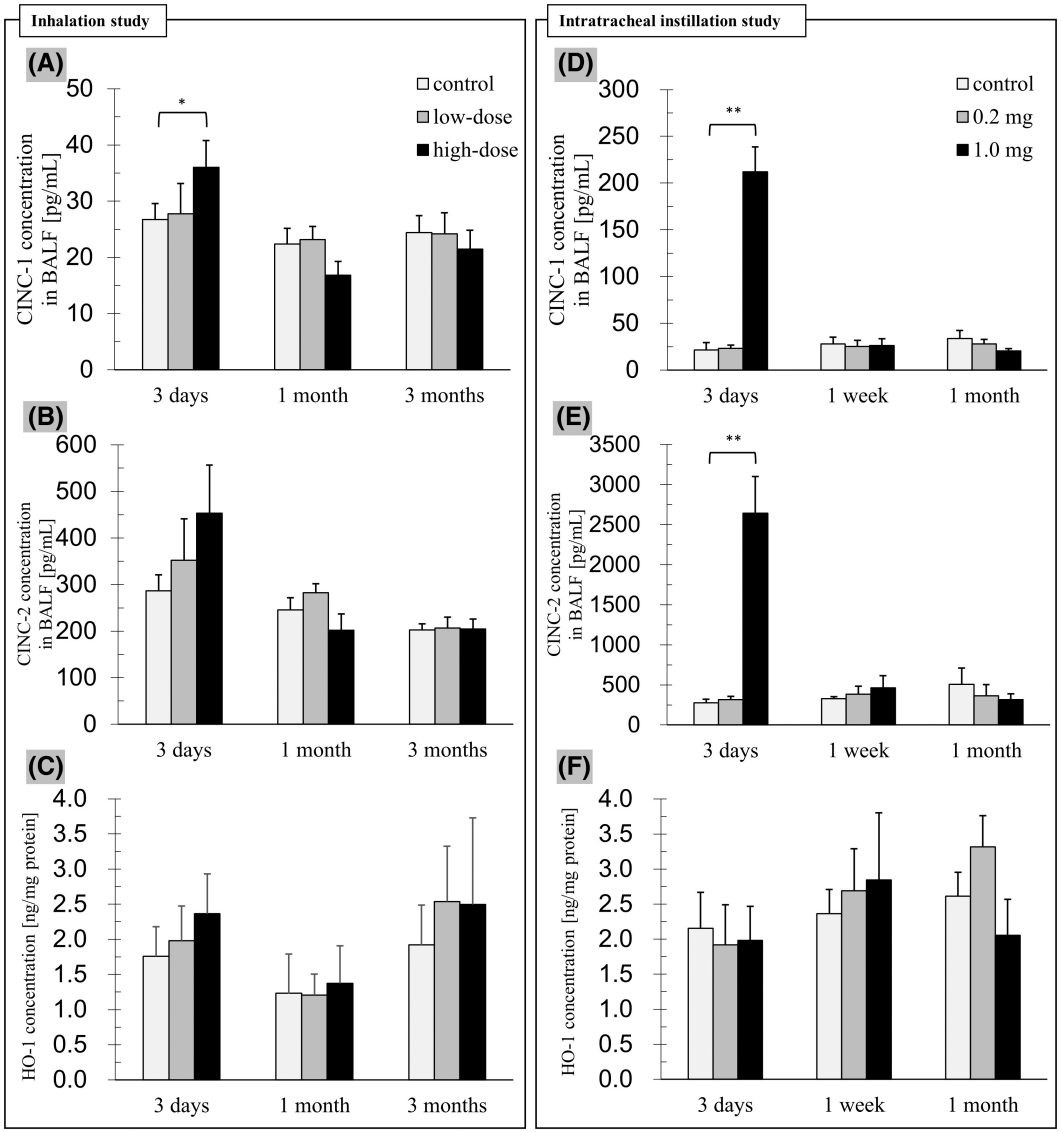
The CINC‐1 and CINC‐2 concentrations in the BALF, and the HO‐1 concentration in the lung tissue. Asterisks mean significant differences compared with the control groups (*P < .05, **P < .01). In both the inhalation (A, B) and intratracheal instillation (D, E) studies, the CINC concentrations in the high‐dose groups increased significantly or had a tendency of an increase at 3 days, but not at the other time points. In both the inhalation (C) and intratracheal instillation (F) studies, there were no statistically significant differences in the HO‐1 concentration between all of the groups at each time point.

Inhalation study: CINC‐1 concentration in the BALF (Figure 4A) was significantly higher in the high‐dose group sacrificed at 3 days after the inhalation compared to the control group (*P* = .023), although not at 1 and 3 months. There were no significant increases between the control group and the low‐dose group at any of the time points. On the other hand, there were no significant differences in the CINC‐2 concentration in the BALF (Figure 4B) between all the groups during the observation period, although there was a tendency of an increase in the high‐dose group at 3 days (*P* = .051). No significant increases were observed between the control group and the low‐dose group at any of the time points.

Intratracheal instillation study: In comparison with the control group, there were statistically significant increases in the concentrations of CINC‐1 (Figure 4D) and CINC‐2 (Figure 4E) in the BALF in the high‐dose group at 3 days after the administration (both: *P* < .001), but not at 1 week or at 1 month.

### 3.4 HO‐1 concentration in lung tissue

Inhalation study: There were no significant differences in the HO‐1 concentration (Figure 4C) between all the groups in every phase.

Intratracheal instillation study: As in the results of the inhalation study, there were no statistically significant differences in the HO‐1 concentration (Figure 4F) between any of the groups at any of the time points.

### 3.5 Correlation between chemokines and neutrophils in the BALF

**FIGURE 5:**
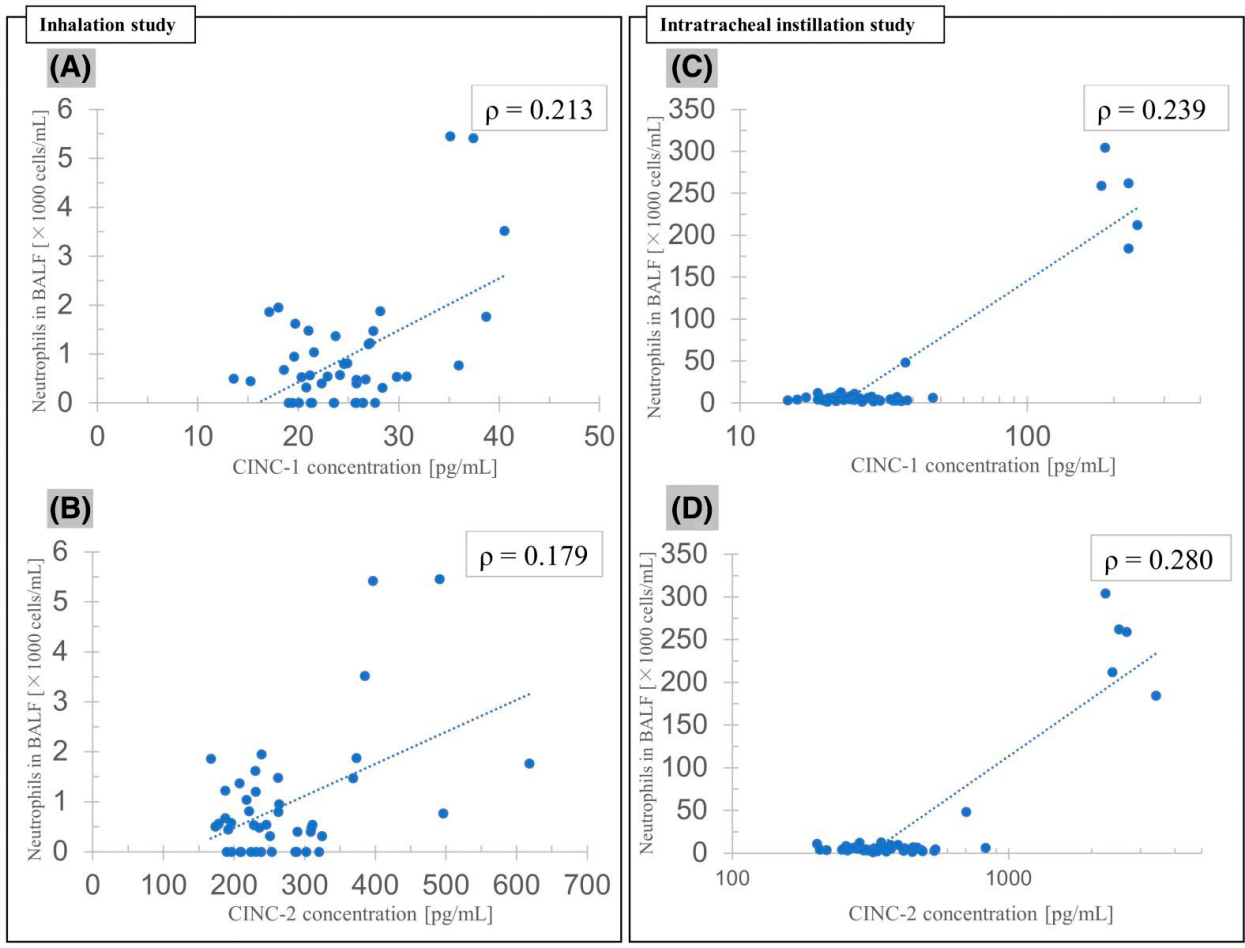
The correlation between CINCs and neutrophils in BALF. In both the inhalation (A, B) and intratracheal instillation (C, D) studies, there were no statistically significant correlations between CINC‐1 and neutrophils or between CINC‐2 and neutrophils.

Inhalation study: Spearman's rank correlation coefficient test showed no statistical correlation between CINC‐1 and neutrophils (Figure 5A) (*P* = .160, *ρ* = .213) or between CINC‐2 and neutrophils (Figure 5B) (*P* = .240, *ρ* = .179).

Intratracheal instillation study: There were no statistical correlations between CINC‐1 and neutrophils (Figure 5C) (*P* = .114, *ρ* = .239) and between CINC‐2 and neutrophils (Figure 5D) (*P* = .062, *ρ* = .280) in the intratracheal instillation study.

### 3.6 Histopathological findings in the lung tissue

**FIGURE 6:**
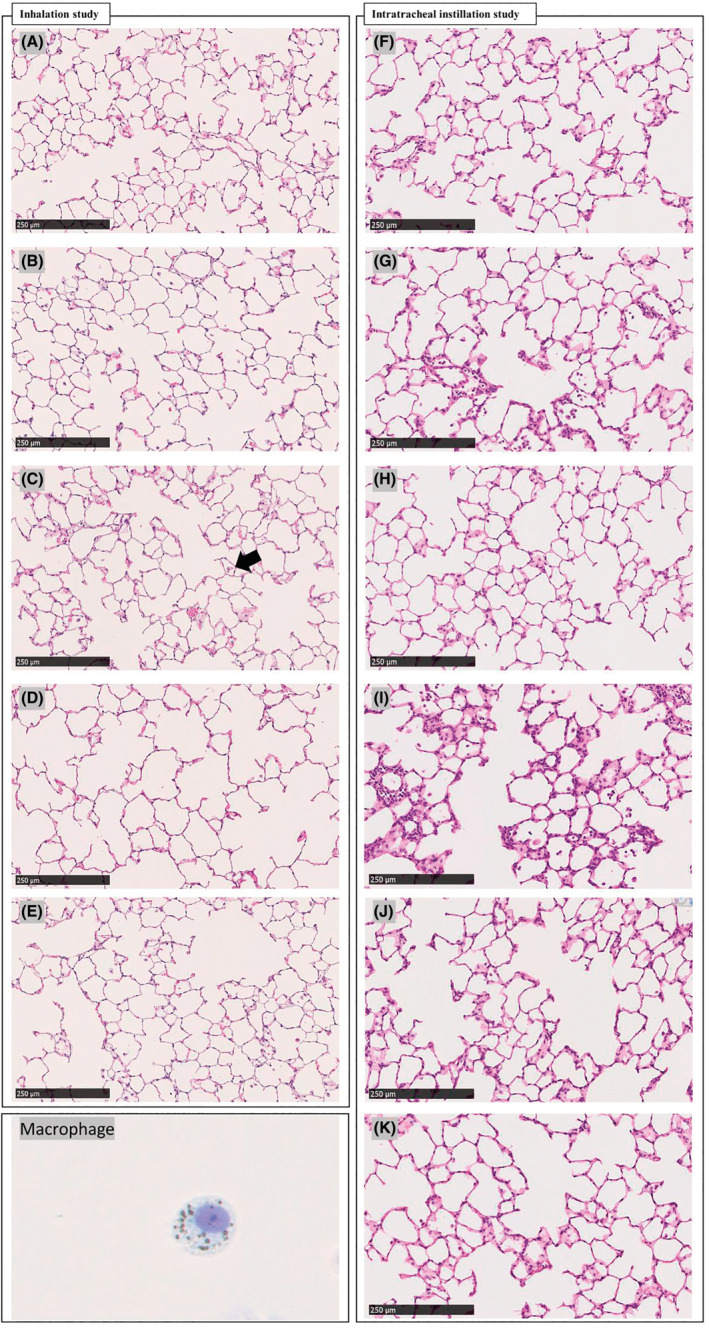
The histopathological observation of the lung sections stained with hematoxylin and eosin. (A)–(E) in the inhalation study shows the following: (A) the control group at 3 days after the inhalation, (B) the low‐dose group at 3 days, (C) the high‐dose group at 3 days, (D) the control group at 3 months, and (E) the high‐dose group at 3 months. Small foam macrophages (shown by the arrow) stood out a little in the high‐dose groups at 3 days after exposure, but no phagocytosed substance was observed in the macrophages, and there was no phagocytosis, inflammation, fibrosis, or neoplastic change in any of the groups not only at 3 days but also at 1 month and 3 months. (F)–(K) in the intratracheal instillation: (F) the control group at 3 days after the intratracheal instillation, (G) the high‐dose group at 3 days, (H) the control group at 1 week, (I) the high‐dose group at 1 week, (J) the control group at 1 month, and (K) the high‐dose group at 1 month. In the high‐dose group at 1 week after the intratracheal instillation, inflammatory cell infiltration composed mainly of macrophages was observed, but little neutrophil infiltration. There was no persistent inflammation, fibrosis, or neoplastic change in any of the groups in either the inhalation or the intratracheal instillation study. The macrophage in BALF is also shown.

Inhalation study (Figure 6A–E): Small foam macrophages stood out a little in the high‐dose groups at 3 days after exposure, but no phagocytosed substance was observed in the macrophages, and there was no phagocytosis, inflammation, fibrosis, or neoplastic change in any of the groups not only at 3 days but also at 1 and 3 months.

Intratracheal instillation study (Figure 6F–K): There were no histopathological findings in any of the groups at 3 days after the intratracheal instillation. Inflammatory cell infiltration composed mainly of alveolar macrophages was observed in the high‐dose group at 1 week, but there was little neutrophil infiltration, and no fibrosis or tumor. No notable change was confirmed in any of the groups during 1 month of observation.

## 4 DISCUSSION

WO_3_ nanoparticles were used as nanomaterials in the present study. Among tungsten particles, tungsten carbide is known to cause hard metal lung disease, which is pathologically giant cell interstitial pneumonia.^9^ The tungsten particles used in the present study were different from tungsten carbide. There are few studies of the toxicity of WO_3_ particles. A 28‐day inhalation study of tungsten blue oxide particles, one kind of WO_3_ particle, was conducted, and the inhalation exposure induced a dose‐related increase in alveolar pigmented and foamy macrophages, but the pulmonary toxicity of the WO_3_ nanoparticles has not been fully clarified.^10^

We performed an inhalation and intratracheal instillation study in order to examine the pulmonary inflammogenicity of WO_3_ nanoparticles. Inhalation studies are thought to be the gold standard study because the style of exposure to the substance is similar to that in humans, and the pattern and amount of lung burden is relatively physiological.^11^, ^12^ For that reason, the response in the inhalation study was emphasized as a physiological response to WO_3_ nanoparticles. On the other hand, although an intratracheal instillation study is a non‐physiological method in which chemicals are injected directly through the trachea, it is possible to administer relatively large doses into animals, if the size of the agglomeration of chemicals is maintained at the respirable level.^13^, ^14^

We adopted 1 mg/rat as the maximum dose in the intratracheal instillation study, because doses of more than 1 mg/rat of any chemical, even chemicals with low toxicity, following intratracheal instillation is thought to induce an excessive response.^15^, ^16^, ^17^ We also previously examined the biopersistence of titanium dioxide (TiO_2_) nanoparticles, chemicals with low toxicity among nanomaterials, in rat lung in an intratracheal instillation study, and the clearance of TiO_2_ nanoparticles in the rat lung began to delay at doses exceeding 1 mg/rat, and inflammation began to occur at the same dose.^18^ Because this inflammation was caused not by the toxicity of the chemical itself but because of the excessive dose, we considered the dose of 1 mg/rat as the maximum dose to avoid excessive response by chemicals in the rat lung.

In the present study, 4 week's inhalation exposure to WO_3_ nanoparticles induced transient neutrophil‐predominant inflammation, judging comprehensively from the BALF analyses and the histopathological features. According to other research about the effects of nanoparticles on the lung under identical experimental conditions, while nanoparticles with high toxicity cause persistent inflammation, and an increase in neutrophils in BALF analysis or neutrophil infiltration in histopathological features, nanoparticles with low toxicity bring out transient inflammation.^19^ Persistent inflammation of neutrophils has been implicated in nanoparticle‐induced lung disorders, and Tomonaga et al.^20^ reported that in an animal study with some nanomaterials with varieties of toxicity to lung, a persistent increase in myeloperoxidase released from neutrophils was correlated with inflammatory cell infiltration, lung damage analyzed from the increase of LDH release, and oxidative stress in lung. Regarding pulmonary inflammation in a 4 week inhalation study of nanoparticles that was the same as in the present study, Morimoto et al.^14^, ^21^, ^22^ reported that nickel oxide (NiO) and cerium dioxide (CeO_2_) nanoparticles, nanoparticles with high toxicities, caused a significant increase in the ratio of neutrophils in BALF for 3 months and inflammatory cell influx consisting mainly of macrophages for 3 months, respectively, whereas TiO_2_ and zinc oxide (ZnO) nanoparticles, nanoparticles with low toxicities, gave rise to only transient inflammation, respectively. A possible reason for the transient inflammation was that the particles were soluble or less cytotoxic and cleared relatively smoothly. We and Adamcakova‐Dodd^22^, ^23^ previously reported that inhalation exposure to soluble ZnO nanoparticles induced transient pulmonary inflammation in rats. On the other hand, we thought that chemical substances with high toxicity to the lung were related to persistent inflammation in the lung because the substance was not cleared significantly from the lung and remained there. IARC^24^ reported that a more biopersistent fiber will have a greater potential to cause lung effects, such as lung fibrosis or thoracic tumors.

In the high concentration groups in the inhalation study, the average of the ratio of neutrophils to all the cells in the BALF at 3 days was 5.8%. The ratios of differential white blood total cell count in the BALF of rats were as follows: that of alveolar macrophages was 91%–100%; neutrophils was 0%–2%; eosinophils was 0%–2%; and lymphocytes was 0%–5%, respectively.^25^ Taking these data into account, although the average of the ratio of neutrophils in the BALF at 3 days in the inhalation study was higher than the normal limits, the rate of increase remained mild. On the other hand, no inflammatory changes were observed as pathological features. Erdely et al.^26^ reported that analysis of BALF was superior to pathological analysis for the sensitivity to detecting pulmonary inflammation in inhalation studies. Moreover, in our previous animal study of cerium dioxide nanoparticles, the pathologist observed few neutrophils in the alveolar space, while there was an increase of neutrophils in the BALF.^21^ We thought that there was a difference between pathological findings and BALF cell analysis in their sensitivity to detecting inflammation. Considering the data in the BALF and pathology, the inflammation brought about by the inhalation of the high‐dose nanoparticles might have been transient and mild.

We also performed an intratracheal instillation study, and WO_3_ nanoparticles did not induce persistent inflammation in rat lung even at the high dose of 1 mg/rat. We used the following formula to estimate what amount in human exposure corresponds to the intratracheal instillation dose (1 mg) in rat.^17^

$$\begin{array}{c} \left(\text{Deposited mass of particles}\right)=\left(\text{exposure concentration of particles}\right)\\ \times \left(\text{tidal volume}\right)\times \left(\text{breathing frequency}\right)\times \left(\text{exposure hours in}a\mathrm{day}\right)\\ \times \left(\text{days of exposure}\right)\times \left(\text{particle deposition fraction}\right). \end{array}$$

Assuming that inhaled chemicals would be deposited at the same rate (particle deposition efficiency 0.1, amount of deposited material/1 g of lung weight) in rats and humans, we estimated that the exposure time per human would be 463 days (calculation in rat and human under assumption of tidal volume 2.1 and 625 ml/times; breathing frequency volume 102 and 12 times/min; exposure hours in day 8 h), if 1 mg/rat as the lung burden was converted into human exposure at a concentration of 3 mg/m^3^, which the American Conference of Governmental Industrial Hygienists (ACGIH) defined as the threshold limit values‐time weighted average (TLV‐TWA) of respirable dust.^27^ We thought that 1 mg/rat as the lung burden of inhaled material by intratracheal instillation might correspond to approximately 1.8 years of inhalation exposure for humans at a concentration of 3 mg/m^3^ (working time 8 h/day, 5 days/week). The dosage given to rats in the intratracheal instillation study, when extrapolated to humans, corresponded to long‐term exposure for workers. Considering that the pulmonary inflammation was transient, and that the dose was equivalent to long‐term exposure for humans, the pulmonary toxicity of WO_3_ nanoparticles was thought to be low not only in the inhalation but also in the intratracheal instillation studies.

Among chemokines, CINCs are the main chemokines for neutrophils in rodents and are related to an influx and activation of neutrophils.^28^ In the present inhalation and intratracheal instillation studies, there was no significant correlation between CINC concentration and neutrophil count in the BALF, although the concentration of CINCs and the neutrophil count in the BALF were thought to be correlated because CINC is a chemokine for neutrophils. Tomonaga et al. reported in analyses of inhalation and instillation studies of NiO, CeO_2_, TiO_2_, and ZnO nanoparticles that there was a significant correlation between CINCs and neutrophils, and CINCs were related to neutrophil influx.^28^ The lack of correlation between the CINC and neutrophil values in our study was likely due to the mild and low inflammation and the relatively wide variability between the samples. The CINC concentrations and neutrophil counts in the BALF in the exposure groups were approximately equivalent to those in the control group.

Some reports^3^, ^29^ have suggested that lung disorders caused by nanoparticles are related to oxidative stress and a gene expression of HO‐1. Inhalation and intratracheal instillation of WO_3_ nanoparticles did not induce a concentration of HO‐1 in lung tissue. Nanoparticles with high toxicities to the lung induce inflammation on the lung and enhance HO‐1 persistently, whereas, in contrast, nanoparticles with low toxicities to the lung induce transient inflammation at most and promote HO‐1 transiently at most.^14^, ^21^, ^22^ Considering the expression of HO‐1 in the present study, we suggest that WO_3_ nanoparticles have low toxicity.

To summarize, in order to investigate the toxicity of WO_3_ nanoparticles on the lung, we conducted an inhalation study and an intratracheal instillation study of WO_3_ nanoparticles to evaluate the inflammatory responses in rat lung. In both studies, exposure to WO_3_ did not induce persistent pulmonary inflammation in rat, suggesting that WO_3_ nanoparticles have low toxicity to the lung. According to the results of the inhalation study, we also propose that the no observed adverse effect level (NOAEL) is 2 mg/m^3^.

## AUTHOR CONTRIBUTIONS

Takashi Marui, Taisuke Tomonaga, Hiroto Izumi, Hidenori Higashi, and Yasuo Morimoto are responsible for the study design and writing of the manuscript; Takashi Marui, Taisuke Tomonaga, and Yasuo Morimoto are responsible for data and analysis; Takashi Marui, Taisuke Tomonaga, Yukiko Yoshiura, Chinatsu Nishida, Ke‐Yong Wang, Miyako Shijo, Masaru Kubo, and Manabu Shimada performed the experiments. All the authors read and approved the final manuscript.

## DISCLOSURE

*Approval of the Research Protocol*: N/A. *Informed Consent*: N/A. *Registry and the Registration No. of the Study/Trial*: N/A. *Animal studies*: All procedures, including animal handling, were done in accordance with the guidelines described in the Japanese Guide for the Care and Use of Laboratory Animals as approved by the Animal Care and Use Committee, University of Occupational and Environmental Health, Japan. *Conflict of Interest*: The WO_3_ nanoparticles were provided by Toshiba Materials Co., Ltd., and the funding for these studies was provided by Toshiba Materials Co., Ltd. The funding source had no role in the design, practice, or analyses of these studies.

## Data Availability

The data that support the findings of this study are available from the corresponding author upon reasonable request.
